# Prescriptions and Dispensing

**Published:** 1908-04-25

**Authors:** 


### Therapeutics.

### PRESCRIPTIONS AND DISPENSING.

PILLS (*continued.*)

A few further rules for pill-making may be laid down as well worthy of observance:—

1. Make a note in the prescription book of the excipient used, so that if the pills are repeated they may be of the same appearance and size.

2. For pills over 1 grain in weight use as little excipient as possible; pills under 1 grain should be made up to 1 grain at least.

3. If it is found necessary to divide the dose into two pills instead of giving it in one, note the fact in the prescription book; note also such facts as the necessity of using the equivalent weight of a dried in place of a hydrated salt, and so forth. In case the pills have to be made up by a locum tenens, for example, this will ensure that the patient does not think an error has been made because the pills are apparently different from those previously dispensed.

4. If all the ingredients of a pill are white, do not use a dark excipient, but dispense as a white pill if possible.

5. If the pill is to contain a volatile ingredient, or one which is liable to be decomposed by contact with the air, varnish the pills.

6. Mix all ingredients that are powders well together before adding any excipient or ingredient of a binding nature. Never trust to powders becoming mixed in the process of massing. All dry ingredients should be in the finest powder possible.

The following may be taken as a type of straightforward pill which involves no difficulty in making:—

| | | |
|---|---|---|
| ℞ | Ferri Sulphatis Exsiccati ... ... | gr. xx. |
| | Aloes Barbadensis ... ... | gr. XL. |
| | Pulveris Cinnamomi compositi ... | ʒj. |
| | Excip. ... ... ... ... ... ... | q. s. |
| | Fiant pilulæ ... ... ... | xxxvi. |

A suitable excipient to use here would be syrup of glucose, of which about 50 grains will be required. It is the official Pilula Aloes et Ferri, the amount of glucose ordered in the Pharmacopœia, calculated to the quantities in the above prescription, being "60 grains, or a sufficient quantity." Most of the official formulæ have a liberal allowance of excipient, and there is no difficulty in making the pills with rather less. Other official pills which give useful practice in dispensing simple formulæ are:—Pilula Aloes Socotrinæ, Pilula Aloes et Myrrhæ, Pilula Colocynthidis composita, Pilula Ipecacuanhæ cum Scillâ, Pilula Plumbi cum Opio, Pilula Rhei composita, Pilula Scammonii composita.

It not infrequently happens that a small fraction of a grain of some active ingredient is required in each pill, and the number of pills to be made requires less than a grain of this ingredient to be taken altogether; there may even be a very inconvenient fraction. The difficulty may occur equally in the case of powders. The following is a case of the kind:—

| | | |
|---|---|---|
| ℞ | Strychninæ ... ... ... ... | gr. $\frac{1}{60}$ |
| | Acidi Arseniosi ... ... ... ... | gr. $\frac{1}{50}$ |
| | Ferri Redacti ... ... ... ... | gr. iiss. |

Fiat pilula j. Mitte xxiv.

The ordinary dispensing scales and weights cannot usually be trusted for quantities less than 1 grain; the quantities here required are two-fifths of strychnine and twelve-twenty-fifths of arsenious acid. A good plan is to weigh out 1 grain of strychnine and $1\frac{1}{2}$ grains of sugar of milk. Powder the strychnine, mix it carefully and thoroughly with the milk sugar, and take 1 grain of this mixture for the prescription. Weigh out 6 grains of arsenious acid and $6\frac{1}{2}$ grains of sugar of milk, mix thoroughly, and take 1 grain of this mixture for the prescription. Reduced iron does not bind well, and a little gum tragacanth is desirable to prevent the mass crumbling; for the quantity here prescribed 4 grains of gum tragacanth may be employed, and the mass then made with syrup of glucose; or 2 grains of gum tragacanth with extract of malt to mass. It is a general rule that tragacanth or acacia gums should never be used for pills unless essential, and then only in small quantity, as pills containing much of either of these do not disintegrate at all readily in the stomach.

When it is possible to avoid the addition of an excipient altogether, or only to use one which will be removed again by evaporation, it is, of course, desirable to do so. The following presents such a case:—

| | | |
|---|---|---|
| ℞ | Camphoræ ... ... ... ... ... | gr. j. |
| | Asafetidæ ... ... ... ... ... | gr. j. |
| | Galbani ... ... ... ... ... | gr. j. |
| | Myrrhæ ... ... ... ... ... | gr. j. |

Fiat pilula j. Mitte xxiv.

If the asafetida, galbanum and myrrh are worked well together in a warm mortar with a warm pestle, it will be possible then to incorporate the camphor, previously powdered, without any excipient. If, however, the mass is not quite soft enough, a very small quantity of spirit can be added. The official Pilula Galbani Composita also contains the three gum-resins heated together, but syrup of glucose is here employed as excipient, and the surplus moisture is removed by the heat employed.

In the following pill a little alcohol is all the addition that is necessary for making the mass:—

| | | |
|---|---|---|
| ℞ | Extracti Aloes Barbadensis ... ... | gr. xij. |
| | Pulveris Nucis Vomicæ ... ... | gr. vj. |
| | Mastich ... ... ... ... ... | gr. vj. |

Misce fiant pilulæ vj.

Pills containing aloes, either Socotrine or Barbadoes, or extract of aloes, always have a tendency to "fall"—that is to say, to flatten on standing; the tendency being more or less noticeable according to the proportion of aloes. The nature of aloes is sufficient to account for this, since it is so easily softened by heat, and especially by heat with a little moisture. Advantage should be taken of this in making the mass, vigorous working being employed so that the mass becomes well warmed, and the whole operation of massing, cutting, and finishing should be carried through without interruption. A minimum of moisture is then necessary, and pills can be made which are soft enough while being finished, but rapidly become hard on standing.

(*To be continued.*)

